# How protected are populations if transmission relapses? Insights from mathematical modeling and simulation

**DOI:** 10.1186/1475-2875-11-S1-O55

**Published:** 2012-10-15

**Authors:** Cátia Bandeiras, Maria Gabriela Miranda Gomes, Carlos Penha-Gonçalves, Maria Jesus Trovoada, Lígia Antunes Gonçalves, Cláudio Romero Farias Marinho, Francisco Freixo, Lars Hviid, Louise Turner

**Affiliations:** 1 Instituto Gulbenkian de Ciência, Rua da Quinta Grande, 6, 2781-901 Oeiras, Portugal; 2 Centre for Medical Parasitology, Department of International Health, Immunology & Microbiology, Faculty of Health Sciences, University of Copenhagen and Department of Infectious Diseases, Copenhagen University Hospital (Rigshospitalet), CSS Øster Farimagsgade 5, Building 22 & 23, Postbox 2099, 1014 Copenhagen, Denmark

## Background

Malaria control measures have been successful in reducing malaria mortality and morbidity in sub-Saharan Africa in the last decade. In particular, in Principe Island, São Tomé e Príncipe, after 5 years of control measures, *Plasmodium falciparum* incidence has decreased 99% and prevalence measured by slide-positivity rate was below 1% in 2009 [1]. However, this method lacks sensitivity for detection of asymptomatic and sub-patent parasite carriage that has implications on transmission [2]. Furthermore, control measures have the adverse effect of promoting decrease of immunity against the parasite, and a relapse in transmission might therefore have more severe consequences on infected individuals [3].

## Materials and methods

Mathematical models are developed to assess age and time trends on malaria infection and immunity against conserved and variant surface antigens of *P. falciparum.* The effect of interventions and transmission relapse on the dynamics of infection and immunity to markers of transmission intensity, such as the merozoite surface protein-3 (MSP-3) and of protection against disease, like *P. falciparum* erythrocyte membrane protein 1 (PfEMPI) at the population level are studied. To calibrate the model, sero-epidemiological data collected in 2005 and 2008 in Principe island with parasitemia determined by polymerase chain reaction (PCR) are used.

## Results

In 2005, with two years of control measures in Principe Island, *P.falciparum* PCR parasitemia was above 25% and, assuming endemic equilibrium, the basic reproduction number was still above 1. Patterns of seropositivity to conserved and anti-PfEMPI antibodies reflect cumulative exposure with age. Simulations of interventions that reduce the transmission coefficient mirror well the progression of infection and anti-MSP-3 immunity in the population in 2008 and suggest that prevalence of infection decays faster than immunity. However, when transmission relapses occur, the onset of immunity is slower than the increase of infected individuals, posing problems for severity of new infections. The simulated number of variants against PfEMP1 decayed steeply with interventions and the effect on particular variants associated with severe disease could give some clues on future malaria morbidity.

## Conclusions

Based on data from a settlement that has been subject to control measures we explore conditions under which the decay on the diversity of the immune repertoire can increase morbidity if transmission relapses and we aim to propose and inspire strategies to undermine the impact of this issue.
